# Kindness Counts: Prompting Prosocial Behavior in Preadolescents Boosts Peer Acceptance and Well-Being

**DOI:** 10.1371/journal.pone.0051380

**Published:** 2012-12-26

**Authors:** Kristin Layous, S. Katherine Nelson, Eva Oberle, Kimberly A. Schonert-Reichl, Sonja Lyubomirsky

**Affiliations:** 1 Department of Psychology, University of California, Riverside, California, United States of America; 2 Department of Educational and Counseling Psychology and Special Education, University of British Columbia, Vancouver, British Columbia, Canada; George Mason University/Krasnow Institute for Advanced Study, United States of America

## Abstract

At the top of parents’ many wishes is for their children to be happy, to be good, and to be well-liked. Our findings suggest that these goals may not only be compatible but also reciprocal. In a longitudinal experiment conducted in 19 classrooms in Vancouver, 9- to 11-year olds were instructed to perform three acts of kindness (versus visit three places) per week over the course of 4 weeks. Students in both conditions improved in well-being, but students who performed kind acts experienced significantly bigger increases in peer acceptance (or sociometric popularity) than students who visited places. Increasing peer acceptance is a critical goal, as it is related to a variety of important academic and social outcomes, including reduced likelihood of being bullied. Teachers and interventionists can build on this study by introducing intentional prosocial activities into classrooms and recommending that such activities be performed regularly and purposefully.

## Introduction

At the top of parents’ many wishes is for their children to be happy, to be good, and to have positive relationships with others [1]–[2]. Fortunately, research suggests that goals for happiness, prosociality, and popularity may not only be compatible but also reciprocal. Happy people are more likely to engage in prosocial behavior [3]–[4] and have satisfying friendships [5]. Similarly, students who are well-liked by peers (i.e., sociometrically popular) are also helpful, cooperative, and emotionally well-adjusted [6]–[8]. Past studies indicate that the link between happiness and prosociality is bidirectional–not only do happy people have the personal resources to do good for others, but prompting people to engage in prosocial behavior also increases well-being [9]–[12]. Based on this prior research–which is predominantly cross-sectional–we predicted that prompting preadolescents to engage in prosocial behavior will boost not only their happiness but also their popularity.

To our knowledge, this study is the first longitudinal experimental intervention of prosocial behavior in preadolescents (“tweens”), and the first to link a manipulation of a simple helping behavior to increases in sociometric popularity (as assessed by peer reports). To explore whether doing good for others (versus engaging in a simple pleasant activity) over 4 weeks would simultaneously increase happiness and promote positive relationships with peers, we randomly assigned 9- to 11-year-olds either to perform acts of kindness (“kindness”) each week or to keep track of places they visited that week (“whereabouts”).

Although the efficacy of happiness-increasing strategies is better established in adults [13], some interventions have boosted well-being in children and adolescents by encouraging gratitude [14]–[15]. Prompting youth to engage in kind acts, however, may have benefits beyond personal happiness, as prosocial behavior predicts academic achievement and social acceptance in adolescents [16]. The dearth of work on enhancing happiness and prosociality in youth, coupled with evidence of their many benefits, highlights the desirability of extending research to this age group.

We predicted that committing kind acts (e.g., carrying groceries) and tracking whereabouts (e.g., visiting grandma’s house or the mall) would both be rewarding activities that would increase well-being in preadolescents. Indeed, the whereabouts task was designed to be a mildly pleasant and distracting control activity (for similar mood-boosting benefits of such activities, see [17]–[18]). For ethical and pragmatic reasons, we wanted to avoid potential harm or waste by not administering the types of “neutral” activities previously used as control tasks (e.g., listing daily hassles or writing essays), which preadolescents may find boring, pointless, or even unpleasant. We also wanted to include a mildly positive comparison group to rule out the possibility that doing kindness increases popularity merely because it feels good. Accordingly, we expected students who practice kind acts–an activity that promotes positive relationships–to experience increases in peer acceptance in addition to increases in well-being. Distinct from other animals, humans as young as 18 months eagerly engage in altruistic acts [19], suggesting that prosociality has a unique evolutionary advantage for human social behavior. Indeed, prosocial behavior has a strong positive association with later peer acceptance [16], and this relationship is likely bidirectional, as children who feel accepted are more likely to do things for others [20], and, in turn, children who do things for others might gain the acceptance of their peers. This latter path has not been studied experimentally. Increasing peer acceptance is a critical goal, as it is related to a variety of important academic [21] and social [22] outcomes, including reduced likelihood of being bullied [23].

## Method

Consent forms describing the study were sent home with students and signed by their guardians. If students brought back a signed consent form, they were then given their own consent form to sign during the researchers’ first classroom visit. The student consent form was verbally explained to the students by the researchers and then students provided written consent. Consent from guardians and students were recorded on a class roster. Only if both guardian and student gave consent was the student given baseline measures. The consent procedure and all data collection were approved by the University of British Columbia’s Behavioral Research Ethics Board (H11-00271) and the Vancouver School Board Ethics Committee.

Nineteen classrooms in the Vancouver, BC school district were randomly assigned to one of two conditions in the second half of the school year. Every week over the course of 4 weeks, students (N = 415, M_age_ = 10.6), nested within classrooms, were instructed either to perform 3 acts of kindness (for anyone they wish) or visit 3 places (anywhere they wish). Throughout the 4-week intervention, students in both conditions reported what they did each week on in-class surveys. Examples of kind acts included “gave my mom a hug when she was stressed by her job,” “gave someone some of my lunch,” and “vacuumed the floor.” Examples of locations visited included “shopping centre,” “baseball diamond,” and “grandma’s house.” All students were told the study was about children’s experiences and emotions.

Before and after the intervention, students reported their life satisfaction (Satisfaction With Life Scale adapted for children; [24]), happiness (Subjective Happiness Scale adapted for children; [25]), and positive affect (child version of the Positive and Negative Affect Schedule; [26]). In addition to the self-report measures, students were provided with a roster of their classmates and asked to circle students (fellow participants) who they “would like to be in school activities [i.e., spend time] with” (a measure of peer acceptance). Students were instructed that they could circle as many or as few names as they liked. At posttest, students were presented with a blank list of their classmates, so they made their new nominations from scratch. Because the study was conducted during the latter half of the school year, students in each classroom already knew each other and were relatively unlikely to continue to make new friends spontaneously. Pre-post changes in self-reports and peer nominations were analyzed using multilevel modeling to account for students’ nesting within classrooms. No baseline condition differences were found on any outcome variables. Further details about method and results are available from the first author.

## Results

Consistent with previous research, overall, students in both the kindness and whereabouts groups showed significant increases in positive affect (γ_00_ = 0.15, S.E. = 0.04, t_(17)_ = 3.66, p<.001) and marginally significant increases in life satisfaction (γ_00_ = 0.09, S.E. = 0.05, t_(17)_ = 1.73, p = .08) and happiness (γ_00_ = 0.11, S.E. = 0.08, t_(17)_ = 1.50, p = .13). No significant differences were detected between the kindness and whereabouts groups on any of these variables (all ps>.18). Results of t-tests mirrored these analyses, with both groups independently demonstrating increases in positive affect, happiness, and life satisfaction (all ts>1.67, all ps<.10).

All students increased in the raw number of peer nominations they received from classmates (γ_00_ = 0.68, S.E. = 0.27, t_(17)_ = 2.37, p = .02), but those who performed kind acts (M = +1.57; SD = 1.90) increased significantly more than those who visited places (M = +0.71; SD = 2.17), γ_01_ = 0.83, S.E. = 0.39, t_(17)_ = 2.10, p = .05, gaining an average of 1.5 friends. The model excluded a nonsignificant term controlling for classroom size (*p* = .12), which did not affect the significance of the kindness term. The effects of changes in life satisfaction, happiness, and positive affect on peer acceptance were tested in subsequent models and all found to be nonsignificant (all *p*s>.54). When controlling for changes in well-being, the effect of the kindness condition on peer acceptance remained significant. Hence, changes in well-being did not predict changes in peer acceptance, and the effect of performing acts of kindness on peer acceptance was over and above the effect of changes in well-being.

## Discussion

Our study demonstrates that doing good for others benefits the givers, earning them not only improved well-being but also popularity. Considering the importance of happiness [27]–[28] and peer acceptance in youth [21]–[22], it is noteworthy that we succeeded in increasing both among preadolescents through a simple prosocial activity. Similar to being happy [29], being well-liked by classmates has ramifications not only for the individual, but also for the community at large. For example, well-liked preadolescents exhibit more inclusive behaviors and less externalizing behaviors (i.e., less bullying) as teens [20]. Thus, encouraging prosocial activities may have ripple effects beyond increasing the happiness and popularity of the doers (cf. [30]). Furthermore, classrooms with an even distribution of popularity (i.e., no favorite children and no marginalized children) show better average mental health than stratified classrooms [8], suggesting that entire classrooms practicing prosocial behavior may reap benefits, as the liking of all classmates soars. Teachers and interventionists can build on our work by introducing intentional prosocial activities into classrooms and recommending that such activities be performed regularly and purposefully.
